# Social Support and Response to AIDS and Severe Acute Respiratory Syndrome

**DOI:** 10.3201/eid1405.071070

**Published:** 2008-05

**Authors:** Arijit Nandi, Melissa Tracy, Allison Aiello, Don C. Des Jarlais, Sandro Galea

**Affiliations:** * Johns Hopkins Bloomberg School of Public Health, Baltimore, Maryland, USA; † University of Michigan School of Public Health, Ann Arbor, Michigan, USA; ‡ Beth Israel Medical Center, New York, New York, USA; § New York Academy of Medicine, New York, New York, USA; ¶ Columbia University Mailman School of Public Health, New York, New York, USA

**Keywords:** Infectious disease, communicable disease, AIDS, SARS, social support, social networks, dispatch

## Abstract

Negative public reactions to emerging infectious diseases can adversely affect population health. We assessed whether social support was associated with knowledge of, worry about, and attitudes towards AIDS and severe acute respiratory syndrome. Our findings suggest that social support may be central to our understanding of public responses to emerging infectious diseases.

The ability of public health institutions to effectively manage emerging infectious diseases (EIDs) and mitigate their consequences is partly a function of public reaction to an epidemic. Negative reactions may vary from denial to panic to stigmatization. Denial or minimization of the threat of an EID by the population at risk can impede prevention efforts and increase transmission. Alternatively, an overreaction to the threat of an EID can overwhelm public health systems and resources, divert resources from effective disease control activities, and lead to severe economic losses in areas affected by the disease. Stigmatization can increase problems of persons with the disease and facilitate transmission because persons with or at risk for the disease may avoid seeking healthcare and because governments may attempt to suppress information about EIDs, considering their potentially severe economic consequences. From a public health policy perspective, identifying personal characteristics and resources that predict responses of persons to EIDs is important to improve the ability of the public to learn about, rationally appraise the threat of, and minimize stigmatization of EIDs.

Determinants of a person’s knowledge, worry, and stigma about EIDs are poorly understood. Although the quality of information conveyed to the public by various sources (e.g., healthcare providers, media) and epidemiologic characteristics of the disease (e.g., mode of transmission, case fatality rate) are important, early empirical work suggests that responses to EIDs are influenced by characteristics that include a greater stock of personal resources. We recently reported that less education was associated with being poorly informed about severe acute respiratory syndrome (SARS), very worried about AIDS, and having more stigmatizing attitudes toward AIDS and SARS (*1*,*2*). Social relationships may also be important. Helleringer et al. reported that social interactions among friends, peers, relatives, and community members may influence perceptions of HIV/AIDS risk and serve “as a resource for individuals to learn about and evaluate new behavioral strategies in the face of the epidemic” (*3*).

In this study, we assessed whether social support was independently associated with knowledge of, worry about, and attitudes toward AIDS and SARS in a representative sample of persons living in the New York City metropolitan area. Social support is defined as the functional aspect of social relationships (*4*).

## The Study

Data for this study came from a cohort of persons >18 years of age who were living in the New York City metropolitan area on September 11, 2001. The cohort was recruited through a random digit dial telephone survey from March 25 through June 25, 2002. Contact information was obtained and follow-up interviews were conducted from September 25, 2002, through January 31, 2003, and from September 24, 2003, through February 29, 2004. Data regarding knowledge of, worry about, and attitudes toward AIDS and SARS were collected among a randomly selected subset of participants in the second cohort follow-up. The total sample included in this analysis, for whom data about AIDS, SARS, and social support were incomplete, consisted of 914 persons; response rate for eligible participants was 56%. Each participant received a $10 incentive. Additional details on sampling are provided elsewhere (*5*). The Institutional Review Board of the New York Academy of Medicine reviewed and approved this study.

We collected information about respondent sex, race/ethnicity, age, educational attainment, marital status, and household income at baseline. Social support was assessed by using a 5-item modified version of the Medical Outcomes Study social support scale (*6*); this abbreviated scale has a Cronbach α of 0.90 (*7*). Social support was categorized as low, medium, or high, on the basis of tertiles of support reported in the sample. We assessed respondents’ knowledge of, worry about, and attitudes toward AIDS and SARS (Table 1). Further details on measures are provided elsewhere (*1*,*2*,*5*).

**Table 1:** Measures of knowledge of, worry about, and stigmatization of AIDS and SARS*

*Measure*	*Question and response options*	*Outcome definition*
Knowledge of AIDS/SARS	Have you heard about AIDS (SARS) a great deal, some, not much, or not at all?	Binary: poorly informed (not much or not at all) versus not poorly informed (some or a great deal)
Worry about AIDS/SARS	Are you very, somewhat, or not at all worried about contracting AIDS (SARS)?	Binary: very worried versus not very worried (somewhat or not at all)
Stigmatization of AIDS/SARS	Do you agree strongly, agree somewhat, disagree somewhat, or disagree strongly with the following statements about controlling AIDS (SARS)? Requiring Americans with AIDS (SARS) to wear identification tags The government announcing it will execute persons who knowingly spread AIDS (SARS) Quarantining or separating all persons with AIDS (SARS) from others in the United States Avoiding areas in the United States that are heavily populated by gay men (Chinese) Forcing all gay men (Chinese) to be medically checked for AIDS (SARS) Not allowing gay men (Chinese) to enter the United States	Continuous: a summary stigmatization scale was constructed for each disease by adding responses to each of the 6 stigma questions; Cronbach α was 0.80 for the AIDS stigmatization scale and 0.72 for the SARS stigmatization scale

*SARS, severe acute respiratory syndrome.

We described sociodemographic characteristics and level of social support of the respondents and created multivariable models predicting knowledge of, worry about, and stigmatizing attitudes toward AIDS and SARS, including covariates thought to potentially confound the relationship between social support and each outcome. Analyses were weighted to correct potential selection bias related to the number of household telephones, persons in the household, and oversampling, as well as to make the sample demographically similar to the New York City metropolitan area population according to US Census 2000 estimates. We used SUDAAN software to estimate standard errors and adjust analyses for the weighting (*8*).

Of the 914 participants, 45.4% were male, 54.1% white, 7.6% Asian or other race, 18.5% black, and 19.8% Hispanic (Table 2). In multivariable models controlling for sociodemographic variables (Appendix Table), persons with low or medium levels of social support were significantly more likely than those with high levels of social support to report being poorly informed about AIDS (p = 0.004 for low support , p = 0.038 for medium support), being very worried about AIDS (p = 0.010 for medium support) and SARS (p = 0.010 for medium support), and to express more stigmatizing attitudes toward SARS (p = 0.020 for low support).

**Table 2:** Sociodemographic characteristics of 914 study participants, New York, New York, metropolitan area

Characteristic	No. (%)
Sex	
M	395 (45.4)
F	519 (54.6)
Race/ethnicity	
White	570 (54.1)
Asian/other	71 (7.6)
Black	130 (18.5)
Hispanic	131 (19.8)
Age, y	
18–34	245 (37.3)
35–54	393 (38.2)
>55	267 (24.5)
Education	
Some college	642 (65.4)
High school or equivalent	182 (25.0)
Less than high school	88 (9.6)
Marital status	
Married	403 (53.0)
Divorced/separated/widowed	210 (15.6)
Never married/unmarried couple	295 (31.4)
Income	
>$75,000	259 (33.9)
$40,000–$74,999	214 (27.5)
$20,000–$39,999	157 (23.5)
<$20,000	129 (15.2)
Social support	
Low	28 (30.2)
Medium	272 (30.5)
High	361 (39.3)

## Conclusions

Reporting higher levels of social support was independently associated with greater knowledge of AIDS but not of SARS. Lower levels of social support were associated with more stigmatizing attitudes toward SARS but not AIDS. These patterns may be explained by differences in the epidemiology of AIDS and SARS in New York City. AIDS has been associated with tremendous illness and mortality rates in New York City (*9*,*10*). In contrast, the SARS epidemic was declared globally contained by the time of data collection (*11*). The psychological pathways through which social support influences responses to EIDs may be dependent on the effect of the epidemic on the local population. For example, greater social support may be associated with greater knowledge of AIDS because, relative to SARS, AIDS is more prevalent and persons with greater social support, through their social relationships, may have greater access to information that influences development of knowledge about EIDs.

Social support was inversely associated with worry about both AIDS and SARS, which suggested that association between social support and AIDS and SARS my persist despite differences in the epidemiology of EIDs. These findings are consistent with studies showing that social interactions are associated with perceptions of the risk for HIV/AIDS (*3*,*12*). Several biological and psychological mechanisms have been theorized to mediate the relationship between social support and health (*4*). Self-efficacy, defined as the degree of confidence persons have in their ability to perform specific tasks, is hypothesized to be one of the primary pathways through which social support operates (*4*). Therefore, social support may act as a buffer against worry about EIDs.

This study has several limitations. First, covariates were treated as time-fixed, which may have resulted in residual confounding. Second, social support is a multidimensional construct; specific dimensions may differentially influence knowledge of, worry about, and stigmatizing attitudes toward EIDs. Finally, for a cross-sectional analysis, it is difficult to establish temporality between levels of social support and knowledge of, worry about, and attitudes toward AIDS and SARS. Observed associations between social support and responses to EIDs may be explained by a common unmeasured covariate.

Emerging and reemerging infectious diseases will continue to profoundly affect human health. The ability to effectively mitigate disease consequences will depend, in part, on minimizing negative public responses. We showed that social support may be central to public responses to EIDs. Further research investigating the pathways linking social support to responses to EIDs may inform interventions that help guide public health and official responses to these diseases.

## Supplementary Material

Appendix Table Variables used in multivariable models to assess the relationship between social support and knowledge, worry, and stigmatization of AIDS and SARS in 914 study participants, New York, New York, metropolitan area*
