# Supplementary material for: Social Support and Response to AIDS and Severe Acute Respiratory Syndrome
Source: Emerg Infect Dis. 2008 May;14(5):825–7. doi: 10.3201/eid1405.071070 (PMC2600224; doi:10.3201/eid1405.071070)
Supplement: Appendix Table — Variables used in multivariable models to assess the relationship between social support and knowledge, worry, and stigmatization of AIDS and SARS in 914 study participants, New York, New York, metropolitan area* [file 07-1070_appT-s1.pdf]

Appendix Table. Variables used in multivariable models to assess the relationship between social support and knowledge, worry, and stigmatization of AIDS and SARS in 914 study participants, New York, New York, metropolitan area*

	Poorly informed about AIDS/SARS							
	AIDS				SARS			
Characteristic	β	SE	p value	Wald p value	β	SE	p value	Wald p value
Social support								
Low	3.69	1.28	0.004	0.011	0.69	0.36	0.056	0.109
Medium	2.60	1.25	0.038		0.07	0.38	0.852	
High	0.00	0.00	–		0.00	0.00	–	
Sex								
M	0.00	0.00	–	0.001	0.00	0.00	–	0.119
F	–2.47	0.75	0.001		–0.47	0.30	0.119	
Race/ethnicity								
White	0.00	0.00	–	0.148	0.00	0.00	–	0.114
Asian/other	1.90	0.92	0.039		0.47	0.51	0.352	
Black	0.76	1.00	0.450		0.05	0.42	0.904	
Hispanic	–0.10	0.76	0.897		1.03	0.48	0.032	
Age, y								
18–34	0.00	0.00	–	0.030	0.00	0.00	–	0.009
35–54	–3.35	1.28	0.009		–0.08	0.40	0.832	
≥55	–1.12	0.77	0.143		1.03	0.45	0.022	
Education								
Some college	0.00	0.00	–	0.178	0.00	0.00	–	0.313
High school or equivalent	1.09	0.69	0.114		0.29	0.35	0.396	
Less than high school	1.75	1.06	0.099		0.83	0.55	0.133	
Marital status								
Married	0.00	0.00	–	0.024	0.00	0.00	–	0.482
Divorced/separated/widowed	–1.09	1.13	0.337		0.01	0.39	0.988	
Never married/unmarried couple	–2.34	0.87	0.007		–0.44	0.39	0.262	
Income								
≥$75,000	0.00	0.00	–	0.561	0.00	0.00	–	0.150
$40,000–$74,999	0.97	0.87	0.261		–0.10	0.43	0.813	
$20,000–$39,999	1.12	0.84	0.186		0.71	0.44	0.107	
<$20,000	1.25	1.04	0.229		0.62	0.54	0.252	

	Very worried about AIDS/SARS							
	AIDS				SARS			
Characteristic	β	SE	p value	Wald p value	β	SE	p value	Wald p value
Social support								
Low	1.11	0.81	0.170	0.019	0.46	0.68	0.499	0.022
Medium	2.15	0.83	0.010		1.44	0.56	0.010	
High	0.00	0.00	–		0.00	0.00	–	
Sex								
M	0.00	0.00	–	0.096	0.00	0.00	–	0.012
F	–1.14	0.68	0.096		1.45	0.57	0.012	
Race/ethnicity								
White	0.00	0.00	–	0.053	0.00	0.00	–	0.256
Asian/other	–0.29	1.65	0.858		1.50	0.76	0.049	
Black	1.93	0.72	0.008		0.77	0.60	0.199	
Hispanic	0.97	0.67	0.151		0.78	0.62	0.207	
Age, y								
18–34	0.00	0.00	–	0.016	0.00	0.00	–	0.081
35–54	–0.76	0.65	0.242		0.77	0.47	0.099	
≥55	–3.07	1.07	0.004		1.39	0.65	0.032	
Education								
Some college	0.00	0.00	–	<0.001	0.00	0.00	–	0.219
High school or equivalent	2.58	0.68	<0.001		–0.80	0.63	0.200	
Less than high school	2.45	0.78	0.002		0.67	0.58	0.250	
Marital status								
Married	0.00	0.00	–	0.268	0.00	0.00	–	0.541
Divorced/separated/widowed	0.99	0.71	0.162		–0.79	0.72	0.271	
Never married/unmarried couple	–0.13	0.71	0.860		–0.26	0.45	0.563	
Income								
≥$75,000	0.00	0.00	–	0.002	0.00	0.00	–	0.274
$40,000–$74,999	0.49	0.84	0.561		–1.34	0.69	0.052	
$20,000–$39,999	–1.21	1.00	0.227		0.08	0.57	0.892	
<$20,000	1.60	0.79	0.043		–0.21	0.60	0.727	

	AIDS/SARS stigmatization scale score†							
	AIDS				SARS			
Characteristic	β	SE	p value	Wald p value	β	SE	p value	Wald p value
Social support								
Low	0.95	0.58	0.100	0.259	1.27	0.55	0.020	0.052
Medium	0.28	0.43	0.512		0.06	0.47	0.894	
High	0.00	0.00	–		0.00	0.00	–	
Sex								
M	0.00	0.00	–	0.138	0.00	0.00	–	0.960
F	−0.64	0.43	0.138		0.02	0.45	0.960	
Race/ethnicity								
White	0.00	0.00	–	0.015	0.00	0.00	–	0.133
Asian/other	2.39	0.98	0.015		0.70	1.05	0.505	
Black	1.37	0.63	0.029		0.98	0.56	0.083	
Hispanic	1.21	0.63	0.057		1.42	0.77	0.066	
Age, y								
18–34	0.00	0.00	–	0.001	0.00	0.00	–	0.110
35–54	−0.52	0.51	0.309		−0.41	0.56	0.469	
≥55	1.48	0.64	0.021		0.62	0.62	0.322	
Education								
Some college	0.00	0.00	–	<0.001	0.00	0.00	–	0.041
High school or equivalent	2.42	0.54	<0.001		1.29	0.55	0.019	
Less than high school	1.87	0.97	0.054		−0.15	1.12	0.897	
Marital status								
Married	0.00	0.00	–	<0.001	0.00	0.00	–	0.021
Divorced/separated/widowed	−1.27	0.55	0.022		0.01	0.61	0.989	
Never married/unmarried couple	−2.08	0.52	<0.001		−1.59	0.59	0.008	
Income								
≥$75,000	0.00	0.00	–	0.100	0.00	0.00	–	0.930
$40,000–$74,999	0.84	0.50	0.091		0.19	0.54	0.732	
$20,000–$39,999	1.20	0.57	0.037		−0.22	0.62	0.720	
<$20,000	1.40	0.83	0.093		0.15	0.90	0.870	

*SARS, severe acute respiratory syndrome; SE, standard error.
†Among those who had heard at least something about AIDS and SARS, respectively.
